# Dr. John H. Sackrider

**Published:** 1911-05

**Authors:** 


Dr. John H. Sackrider, of East Randolph, N. Y., died at his home March 12, 1911, from neuritis, aged 63 years. He graduated from the University of Buffalo in 1878.

